# Neurobehavioral Symptoms in Community-Dwelling Adults With and Without Chronic Traumatic Brain Injury: Differences by Age, Gender, Education, and Health Condition

**DOI:** 10.3389/fneur.2019.01210

**Published:** 2019-11-20

**Authors:** Shannon B. Juengst, Andrew Nabasny, Lauren Terhorst

**Affiliations:** ^1^Department of Physical Medicine & Rehabilitation, University of Texas Southwestern Medical Center, Dallas, TX, United States; ^2^Department of Occupational Therapy, University of Pittsburgh, Pittsburgh, PA, United States

**Keywords:** traumatic brain injury, behavior, emotion, gender, age, education

## Abstract

Neurobehavioral symptoms after Traumatic Brain Injury (TBI) are prevalent, persist for many years, and negatively affect long-term health, function, and quality of life. Symptoms may differ based on age, gender, education, race, ethnicity, and injury severity. To better understand neurobehavioral functioning after TBI, we need a comprehensive picture of emotional, cognitive, and behavioral symptoms in the context of personal factors that may affect these symptoms. We also need to understand the extent to which these symptoms are specific to TBI, shared across other neurological conditions, or attributable to factors outside of the injury itself. We collected neurobehavioral symptoms via the self-reported Behavioral Assessment Screening Tool (BAST) in a National Cohort of English (*n* = 2,511) and Spanish speaking (*n* = 350) community-dwelling adults with and without chronic TBI and other neurological and mental health conditions. The primary focus of the present study was to comprehensively describe neurobehavioral symptoms in adults with and without TBI, broken down by gender and health conditions and then further by age group or educational attainment. As expected, participants with TBI reported more symptoms than Healthy Controls. Regardless of condition, women reported more fatigue, while men reported more substance abuse and impulsivity. Hispanic participants reported more neurobehavioral symptoms than non-Hispanic participants did across health conditions, though primarily Spanish-speakers reported fewer symptoms than English-speakers, suggesting that level of acculturation may contribute to symptom reporting. These data provide a comprehensive characterization of neurobehavioral symptoms in adults with TBI and adults without TBI (healthy controls, adults with other neurological conditions, and adults with mental health conditions).

## Introduction

Following Traumatic Brain Injury (TBI), emotional and behavioral symptoms are prevalent, often persist for many years, and negatively affect long-term health, function, and quality of life (1–7). Neurobehavioral symptoms, including aggression, disinhibition, lack of motivation, and planning/executing actions (8, 9), are among the greatest contributing factors to poor outcomes after TBI (e.g., disability, depression, suicidality, quality of life) (5, 10, 11). The extent to which neurobehavioral symptoms in chronic TBI result from the injury itself or are due to other factors, such as personal characteristics or comorbid neurological or mental conditions remains unclear. Neurobehavioral symptoms are more likely to occur in the context of chronic emotional disruptions [e.g., depression (12–15)] that are common in the general population and after TBI (16, 17). Symptom-reporting after injury may also differ based on age (18, 19), gender (20, 21), education (22), race or ethnicity (23), and injury severity (24, 25), though differences in symptom reporting by these personal factors are present in other clinical populations and the general population as well. A nationwide epidemiological study on TBI-related emergency department visits concluded that many of the common neurobehavioral symptoms after TBI may be more related to pre-existing psychiatric conditions (e.g., post-traumatic stress disorders, substance use disorders) and personality features (e.g., impulse control problems, high-risk behaviors) that put an individual at greater risk for sustaining a TBI, rather than to just the injury itself (17). Similarly, a recent study on chronic traumatic encephalopathy (CTE) found that many of the clinical symptoms used to diagnose CTE, including mood, emotional regulation, and behavioral symptoms, were also common in men with depression in the general population, indicating that these symptoms are not necessarily specific to brain pathology (26). To better understand neurobehavioral functioning after TBI, we need a comprehensive and concurrent picture of emotional, cognitive, and behavioral symptoms in the context of personal factors and health conditions that may affect these symptoms.

### Age

The extent to which age-related differences in neurobehavioral symptoms are specific to TBI or attributable only to age effects remains unclear. Psychiatric symptoms appear to occur more often in younger adults, whereas fatigue and other physiological symptoms appear to be more common in older adults, both in the general population and among those with TBI. One study reported that adults under 65 years old with TBI more often had psychiatric diagnoses compared to those over 65 with TBI (19), consistent with the pattern of psychiatric diagnoses by age observed in the general population (27, 28). Breed et al. (18) compared injury severity-matched older and younger adults with TBI and age-matched healthy controls on a number of self-reported symptoms. They found that younger adults (18–35 years) with TBI reported more sleep difficulties than older adults with TBI (over 55) and age-matched controls. Older adults with TBI reported more fatigue-related symptoms and more neurologic symptoms, such as headaches, seizures, and difficulties producing and understanding speech, than age-matched peers without TBI (18). Despite similar main effects of age on symptom-reporting, experiencing a TBI does seems to exacerbate problems related to sleep and neurologic symptoms.

### Gender

Psychiatric and neurobehavioral symptoms are more prominent in women than men, both in the general population and after TBI. Women report more symptoms of depression, anxiety, fatigue, and, in some studies, sleep disturbances following TBI (20, 21). Men report more restlessness, more difficulty setting realistic goals, and in some studies more sleep disturbances than women after TBI (21). A review of the literature reveals similar patterns within the general population as well, with higher rates of depression and subsequent fatigue and anxiety among women compared to men (29). One study on gender differences in executive function post-TBI found that women had better executive functioning than men, even after controlling for education and ethnicity (30). In the general population, men are more prone to impulsive behaviors (31, 32). but women may be more likely to report impulsivity symptoms when they are present (31). Further, the relationship between impulsivity and substance abuse may differ as a function of gender, with a stronger association among women than men (32). Notably, despite a growing acceptance that gender is not a binary construct, the literature on gender differences after TBI fails to represent genders outside of men and women.

### Education

The relationship between educational attainment and mental health symptoms is complicated. Prior research in the general population in Sweden demonstrated a negative correlation between mental health issues during childhood and ultimate educational attainment (33), suggesting that a predisposition to poorer mental health early in life may negatively impact education level later in life. A study on a nationally representative sample of non-institutionalized adults in the United States found that those with lower levels of education reported higher rates of depressive symptoms, lower rates of treatment, and greater likelihood of increasing depressive symptoms over time (34). Similarly, a longitudinal study by Dikmen et al. (22) found that individuals without a high school degree had greater depressive symptoms than those with at least a high school degree, 1-year post-TBI. The authors suggested that these individuals may have fewer resources to help cope with the injury, as well as greater difficulty in returning to work (22). In both the general population and TBI, the adverse effects of lower education may be more prominent in individuals from disadvantaged vs. advantaged backgrounds (35–37).

### Race and Ethnicity

Racial and ethnic minority groups, particularly Hispanic individuals, are at greater risk for sustaining a TBI (38) and report more psychiatric symptoms, physical limitations, and cognitive deficits following TBI compared to non-Hispanic white individuals (23, 39–41). Racial and ethnic minorities also frequently experience health care disparities (23, 39–41) that magnify the long-term consequences of TBI (37, 39, 41–46). In contrast, Hispanic and black individuals within the general population are less likely than non-Hispanic white individuals to report a psychiatric diagnosis (47). Research on the effects of race and ethnicity on neurobehavioral symptoms is therefore complicated by potential differences in symptom experience vs. differences in symptom reporting.

### TBI Injury Severity: Mild vs. Moderate-Severe

Chronic neurobehavioral symptoms after TBI can occur across all levels of injury severity (1–7). Moderate-to-severe TBI may have a larger physiological and cognitive impact, but greater self-awareness and less obvious disability following a mild TBI, as compared to moderate-to-severe TBI, may lead to more emotional distress and subsequent reporting of stress and depressive symptoms (48). In a study on combat-related TBI, individuals with mild TBI endorsed more posttraumatic stress and post-concussive symptoms than those with moderate-to-severe TBI, even after controlling for age, time since injury, and mechanism of injury (24). However, after controlling for posttraumatic stress symptoms, there were no differences in post-concussive symptoms, leading the authors to suggest that greater post-concussive symptom-reporting was a function of emotional distress rather than injury severity (24). Examining differential patterns in neurobehavioral symptoms as a function of injury severity may improve targeted intervention.

To date, research looking across injury severity and across factors known to impact neurobehavioral symptoms post-TBI is limited. Though Breed et al. compared symptom reporting between older and younger individuals with TBI to each other and to age-matched healthy controls, they focused on physical symptoms rather than neurobehavioral and emotional consequences of brain injury (18). Holzer et al., in a recent epidemiological study in TBI, specifically identify a need for more research to examine the interplay between TBI and both psychiatric and personality-related factors to better understand post-injury neurobehavioral symptoms (17). No one has directly compared neurobehavioral symptoms in healthy controls vs. those with TBI, nor has anyone compared individuals with TBI to individuals with other neurological or mental health conditions commonly co-occurring with TBI that could explain post-injury neurobehavioral symptoms. It is currently unclear if these neurobehavioral symptoms, seen post-TBI, are due to brain injury itself, an increased risk for neurological and/or mental condition(s) predating or following TBI, or a combination of these factors. Furthermore, despite the growing proportion of Spanish-speaking adults in the United States (49), these individuals are often excluded from research on chronic TBI (43).

The current study presents a neurobehavioral characterization from a nationally representative sample of individuals with TBI across levels of injury severity and individuals without TBI, both with and without other neurological or mental health conditions. We used a patient-reported outcome measure of neurobehavioral symptoms, the BAST, to capture multiple dimensions of neurobehavioral function. We examined differences in neurobehavioral symptoms across health condition groups (healthy controls, mild TBI, moderate-severe TBI, history of other neurological conditions, and history of mental health conditions). We then examined neurobehavioral symptoms within each health condition group by gender and age or education. We also explored differences based on ethnicity and primary language. This study thoroughly characterizes neurobehavioral symptoms common after TBI and provides comparative data in both English- and Spanish-speaking healthy controls, individuals with TBI, and individuals with other neurological and/or mental health conditions, both for future studies and for clinical practice.

## Materials and Methods

### Setting

We collected self-reported neurobehavioral symptoms electronically via Qualtrics™ in a nation-wide survey study of community-dwelling adults with and without TBI.

### Participants

Participants were adults (>18 years old), fluent in either English or Spanish, with no self-reported history of schizophrenia or dementia, who electronically consented to participate. For the purposes of this study, we separated those who took the survey in English and those who took the survey in Spanish into two distinct cohorts.

### Procedures

**Figure 1:**
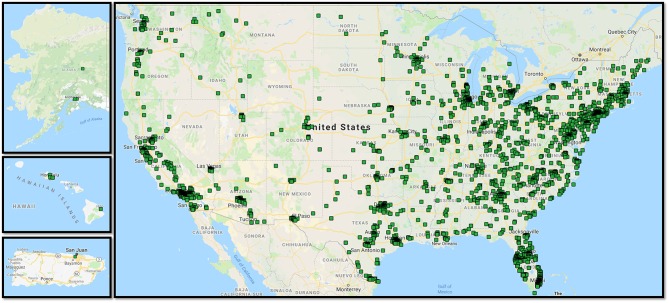
Mapped survey responses for the National cohort study. Map made using BatchGeo (http://batchgeo.com/).

We created an electronic survey of the BAST using Qualtrics™ (Qualtrics, Provo, UT) HIPAA-compliant survey platform, to collect data from a national sample from both the general population and those with self-reported lifetime history of TBI. We established sampling quotas based on: (1) age and gender distributions generally observed in TBI; (2) BAST language (English or Spanish); and (3) presence or absence of self-reported history of TBI or concussion. Qualtrics serves as a survey panel aggregator, leveraging multiple survey companies to send out electronic requests for participants willing to complete a survey study. Through this service, we obtained electronic survey responses meeting our sampling quotas. Qualtrics automatically removed survey responses completed in <1/3 of the median time it took the first 150 participants to complete the survey. Our study team conducted further data checks on all surveys, with Qualtrics removing and replacing surveys deemed invalid by the study team. Determination of invalidity was based on gibberish in open-text responses, illogical responses (e.g., endorsing never feeling fatigue and always limiting physical activities because of fatigue), questionable open-text responses with other evidence that responses were invalid (e.g., duration of the total survey, validity checks, inconsistency in answers), and the two validity items embedded in the BAST. Though we do not have data on how many potential participants received an invitation to take the survey, nor how many Qualtrics automatically removed as a part of their internal validity check, we tracked all survey responses we received and reasons for deeming surveys ineligible. Qualtrics removed and replaced *n* = 558 invalid responses over the course of 12 separate data quality checks conducted by study investigators. Further data quality checks after the survey was close resulted in removal of an additional *n* = 202 invalid responses, the majority (*n* = 164) due to the survey being taken in English by non-fluent native Spanish speakers (open-ended responses provided in Spanish). We confirmed TBI presence and severity with an electronic version of a structured questionnaire modeled after the OSU-TBI (50). Capturing these data via structured electronic questionnaire has demonstrated validity in a prior study in a TBI sample (51). We then generated a map of all responses to show national representation of the study cohort, depicted in Figure 1.

### Measures

A demographic questionnaire captured age, gender, race, ethnicity, highest level of education completed, native language, and history of various health conditions. Based on the health conditions selected, participants were either excluded (dementia, schizophrenia) or categorized as:

- Healthy Controls (selecting “none of the above”)
- Other Neuro Conditions (selecting any of the following: learning disability, ADHD, Stroke, Multiple Sclerosis, Parkinson's Disease, Autoimmune condition, Other neurological condition)
- Mental Health Conditions only (selecting only one or more of the following: Post-traumatic stress disorder, Bipolar disorder, depression, anxiety, alcohol abuse/dependence, drug abuse/dependence, other mental health condition)
- TBI (selecting Traumatic Brain Injury or Concussion). Those categorized in the TBI group also completed a questionnaire following the structure and content of the OSU-TBI, to confirm history of TBI and to classify injury severity. The OSU-TBI Worst Injury Score ranges from 1 to 5, with 1 indicating no history of TBI, 2 and 3 indicating mild TBI, 4 indicating moderate TBI, and 5 indicating severe TBI (50). These scores classified participants' injuries as Mild or Moderate-Severe. This includes any lifetime history of TBI (e.g., during childhood or adulthood).

Participants selected their preferred language for completing the survey (English or Spanish), and all subsequent questionnaires were presented in their preferred language.

#### Behavioral Assessment Screening Tool (BAST)

Effectively measuring neurobehavioral symptoms in community-dwelling populations is challenging, but necessary for valid and clinically relevant characterization and interpretation to inform problem-identification and treatment. The Behavioral Assessment Screening Tool (BAST) is a self-reported neurobehavioral symptom measure developed based on a theoretical model that frames behavior as an overarching concept with multiple interacting domains, including emotions, cognitive function, and personal factors, all in the context of individuals' existing environmental supports and stressors (52). It employs simple language and sentence structure at the 8–9th grade reading level in both English and Spanish, includes items specifically assessing validity of responses, and has a validated multidimensional structure to cover the complexity of neurobehavioral symptoms after TBI (53–55). We developed the Spanish-language version through both translation and language validation, including a language validation committee and cognitive interviewing (55).

The BAST, in both English and Spanish, measures self-reported neurobehavioral symptoms in five domains: Negative Affect, Fatigue, Substance Abuse, Executive Function, and Impulsivity. It demonstrates good content validity and a multidimensional factor structure with good internal consistency reliabilities (Cronbach's alpha's ranging from α = 0.76–0.90) (54) among community-dwelling adults with chronic TBI (53, 54). To create comparable scores for each subscale, we summed the individual item scores and divided by the total number of items within each subscale; this yielded an average subscale score ranging from 1 to 5, with one indicating never experiencing that symptom and 5 indicating experiencing that symptom very often. Of note, though the BAST was developed and initially validated in chronic TBI, none of the symptoms is specific to brain injury, nor is any attribution made as to the etiology of the symptoms. The purpose of the BAST is screening for problematic neurobehavioral symptoms that may negatively affect individuals' health and well-being, regardless of etiology. However, as no one has yet validated the BAST in adults without TBI, we present Cronbach's α for each of the subscales within each cohort as a measure of the BAST's reliability in non-TBI samples. Cronbach's α's can be interpreted as follows: >0.70 = Acceptable, >0.80 = Good, >0.90 = Excellent.

### Analyses

We calculated frequency and percentages to characterize each health condition group and means with standard deviations for each of the BAST subscales to characterize neurobehavioral symptoms. BAST subscale average scores were computed by summing scores on subscale items and dividing by the total number of items, which yielded comparable scores ranging from 1 (never) to 5 (very often) for each subscale. We present descriptive data broken down by gender and health conditions and then further by either age group or educational attainment among primary English speakers and primary Spanish speakers, separately. We also compared BAST subscale scores within each health by ethnicity and primary language. We used non-parametric tests for independent samples (Kruskal-Wallis, Mann-Whitney) for all comparisons. All analyses were performed using Statistical Packages for the Social Sciences (SPSS, v.24) software with a conservative overall significance level of α = 0.01 to account for multiple testing and an adjusted alpha = 0.003 for *post hoc* testing.

## Results

### Participants

After completing all validity checks, we retained 2,511 complete BAST surveys in English (*n* = 2,461 were native English-speakers, 50 were native Spanish-speakers fluent in English), of which *n* = 2,248 reported no TBI history, *n* = 211 reported Mild TBI, and *n* = 52 reported Moderate-Severe TBI. For those with no history of TBI (*n* = 2,248), we further broke down the cohort into those with no self-reported history of neurological or mental health condition; (Healthy Controls; *n* = 1,548), neurologically healthy controls with *only* a self-reported history of a mental health condition (Mental Health Conditions; *n* = 427), and controls who reported *any* history of another neurological condition (Other Neuro Conditions; *n* = 273). Table 1 presents demographic and neurobehavioral symptom data by group for all participants taking the survey in English.

**Table 1:** Demographics and neurobehavioral symptoms in English-speaking community-dwelling adults with and without TBI.

**Participant characteristics**		**English-speaking national cohort (*****n*** **=** **2511)**				
		**Mild TBI** ***n* = 211**	**Moderate-severe TBI** ***n* = 52**	**Healthy controls** ***n* = 1548**	**Mental health conditions** ***n* = 427**	**Other neuro conditions** ***n* = 273**
		***n* (%)**	***n* (%)**	***n* (%)**	***n* (%)**	***n* (%)**
Gender	Women	114 (54.0%)	20 (38.5%)	544 (35.1%)	225 (52.7%)	137 (50.2%)
	Men	92 (43.6%)	30 (57.7%)	998 (64.5%)	196 (45.9%)	130 (47.6%)
	Transgender/other	5 (2.4%)	2 (3.8%)	6 (0.4%)	6 (1.4%)	6 (2.2%)
Race	White	180 (85.3%)	39 (75.0%)	1104 (71.4%)	321 (74.2%)	210 (77.0)%
	Black/African American	11 (5.2%)	4 (7.7%)	181 (11.7%)	43 (10.1%)	23 (8.5%)
	Asian	6 (2.8%)	5 (9.6%)	50 (3.3%)	9 (2.1%)	10 (3.7%)
	American Indian/Alaskan Native	4 (1.9%)	1 (1.9%)	21 (1.4%)	7 (1.6%)	3 (1.1%)
	Native Hawaiian/Pacific Islander	1 (0.5%)	2 (3.8%)	6 (0.4%)	1 (0.2%)	2 (0.7%)
	Other	7 (3.3%)	1 (1.9%)	172 (11.2%)	43 (10.2%)	20 (7.3%)
	Unknown	2 (0.9%)	0 (0%)	13 (0.8%)	3 (0.7%)	5 (1.8%)
Ethnicity	Hispanic	23 (10.9%)	6 (11.5%)	448 (30.0%)	114 (26.7%)	57 (20.9%)
	Non-hispanic	183 (86.7%)	46 (88.5%)	1,046 (67.6%)	307 (71.9%)	209 (76.6%)
	Unknown	5 (2.4%)	0 (0%)	54 (3.5%)	6 (1.4%)	7 (2.6%)
Education	≤High school	37 (17.5%)	9 (17.3%)	466 (30.1%)	158 (37.0%)	112 (41.0%)
	>High school	174 (82.5%)	43 (88.5%)	1,082 (69.9%)	269 (63.0%)	161 (59.0%)
		**Mean (*****SD*****)**	**Mean (*****SD*****)**	**Mean (*****SD*****)**	**Mean (*****SD*****)**	**Mean (*****SD*****)**
Age (years)		40.55 (15.50)	44.15 (15.72)	44.55 (17.73)	39.58 (15.15)	37.45 (14.73)
Range		18–81	21–82	18–90	18–86	18–78
BAST subscales	Negative affect	3.26 (0.75)	3.16 (0.69)	2.55 (0.61)	3.34 (0.68)	3.26 (0.69)
	Fatigue	3.16 (0.89)	3.07 (0.70)	2.40 (0.70)	3.15 (0.80)	3.14 (0.76)
	Executive function	2.27 (0.66)	2.40 (0.68)	2.05 (0.52)	2.32 (0.58)	2.37 (0.57)
	Impulsivity	2.30 (0.75)	2.44 (0.82)	2.04 (0.69)	2.41 (0.81)	2.43 (0.76)
	Substance abuse	1.83 (0.94)	2.10 (1.03)	1.43 (0.67)	1.81 (0.96)	1.72 (0.93)

*All BAST Subscale values are mean (standard deviation) of the average score across items in each subscale*.

*Color values in the tables are to allow for comparison across tables of BAST subscales. Purple, negative affect; Green, fatigue; Blue, executive function; Red, impulsivity; Yellow, substance abuse*.

We retained 350 complete BAST surveys in Spanish, of which *n* = 11 reported a history of Mild-Severe TBI, *n* = 296 were Healthy Controls, *n* = 27 had only Mental Health Conditions, and *n* = 16 had Other Neuro Conditions. **Table 3** presents demographic and neurobehavioral symptom data by group for primarily Spanish-speaking participants.

### Neurobehavioral Symptoms

Neurobehavioral symptoms broken down by gender and condition for each subscale are further broken down by age group or by educational attainment in Table 2 for English-speaking participants. Numbers of participants in each cell of Table 2 are available in Supplemental Table A. For primarily Spanish-speaking participants, Table 3 presents neurobehavioral symptoms separately by condition and Table 4 presents neurobehavioral symptoms broken down by gender or educational attainment within each health condition group.

**Table 2:** Neurobehavioral symptoms by gender, neurological and mental health conditions, age, and education among English-speakers.

**BAST subscale**	**Age (years)**	**Women**					**Men**				
		**Mild TBI**	**Moderate-severe TBI**	**Healthy controls**	**Mental health conditions**	**Other neuro conditions**	**Mild TBI**	**Moderate-severe TBI**	**Healthy controls**	**Mental health conditions**	**Other neuro conditions**
Negative affect	18–24	3.67 (0.57)	3.54 (0.22)	3.01 (0.68)	3.62 (0.65)	3.68 (0.78)	3.13 (0.65)	3.69 (0.20)	2.68 (0.53)	3.35 (0.66)	3.23 (0.64)
	25–45	3.62 (0.72)	3.90 (0.56)	2.70 (0.63)	3.39 (0.59)	3.36 (0.67)	3.13 (0.69)	3.04 (0.43)	2.61 (0.63)	3.31 (0.70)	3.26 (0.62)
	46–65	3.18 (0.70)	3.09 (0.63)	2.56 (0.60)	3.26 (0.67)	3.08 (0.71)	3.04 (0.77)	3.06 (0.98)	2.39 (0.56)	3.18 (0.77)	3.10 (0.65)
	>65	2.62 (0.65)	2.81 (0.16)	2.30 (0.55)	3.25 (0.74)	2.79 (0.54)	2.34 (0.34)	2.67 (0.51)	2.32 (0.51)	2.97 (0.55)	2.62 (0.49)
Fatigue	18–24	3.45 (0.79)	3.08 (0.12)	2.65 (0.70)	3.19 (0.83)	3.60 (0.76)	2.73 (0.68)	3.67 (0.76)	2.53 (0.66)	3.06 (0.71)	2.85 (0.69)
	25–45	3.56 (0.86)	3.93 (0.32)	2.52 (0.73)	3.39 (0.82)	3.32 (0.80)	2.94 (0.75)	2.92 (0.60)	2.40 (0.70)	3.00 (0.81)	2.99 (0.65)
	46–65	3.10 (0.99)	2.78 (0.73)	2.42 (0.75)	3.10 (0.73)	3.16 (0.83)	3.02 (0.92)	2.81 (0.79)	2.23 (0.68)	2.96 (0.90)	3.00 (0.80)
	>65	3.19 (0.64)	3.08 (0.83)	2.29 (0.68)	3.23 (0.69)	3.36 (0.93)	2.43 (0.62)	3.04 (0.34)	2.31 (0.63)	2.95 (0.59)	2.77 (0.50)
Executive function	18–24	2.28 (0.69)	3.36 (0.26)	2.18 (0.50)	2.40 (0.61)	2.73 (0.72)	2.49 (0.67)	2.67 (1.15)	2.20 (0.53)	2.42 (0.65)	2.42 (0.53)
	25–45	2.32 (0.75)	2.94 (0.67)	2.02 (0.53)	2.32 (0.57)	2.40 (0.52)	2.38 (0.68)	2.47 (0.66)	2.13 (0.55)	2.40 (0.57)	2.37 (0.57)
	46–65	2.13 (0.60)	2.04 (0.30)	1.96 (0.47)	2.17 (0.57)	2.22 (0.59)	2.21 (0.58)	2.36 (0.63)	2.00 (0.47)	2.27 (0.59)	2.43 (0.53)
	>65	1.82 (0.30)	1.68 (0.32)	1.81 (0.45)	2.31 (0.47)	2.04 (0.50)	2.24 (0.43)	2.07 (0.34)	1.92 (0.41)	2.20 (0.48)	2.08 (0.52)
Impulsivity	18–24	2.50 (0.70)	2.75 (0.35)	2.19 (0.66)	2.52 (0.60)	2.64 (0.65)	2.61 (0.75)	3.67 (0.72)	2.44 (0.75)	2.85 (0.76)	2.69 (0.67)
	25–45	2.39 (0.77)	2.79 (0.67)	1.99 (0.69)	2.25 (0.77)	2.34 (0.78)	2.56 (0.75)	2.55 (0.88)	2.24 (0.73)	2.63 (0.87)	2.66 (0.81)
	46–65	2.00 (0.71)	2.03 (0.34)	1.76 (0.56)	2.00 (0.59)	1.99 (0.60)	2.04 (0.69)	2.28 (1.00)	1.87 (0.59)	2.38 (0.81)	2.23 (0.59)
	>65	1.50 (0.35)	2.50 (0.35)	1.54 (0.41)	2.23 (0.75)	1.93 (0.40)	1.90 (0.58)	2.13 (0.72)	1.92 (0.52)	2.33 (0.76)	2.16 (0.63)
Substance abuse	18–24	1.81 (0.83)	2.50 (0.71)	1.16 (0.38)	1.58 (0.85)	1.61 (0.99)	1.73 (0.83)	2.78 (0.69)	1.41 (0.64)	1.88 (0.98)	1.73 (1.04)
	25–45	1.76 (0.96)	2.29 (1.45)	1.40 (0.63)	1.55 (0.73)	1.81 (0.93)	2.37 (1.10)	2.55 (1.27)	1.73 (0.81)	2.31 (1.11)	2.00 (1.00)
	46–65	1.39 (0.60)	1.67 (0.58)	1.28 (0.54)	1.48 (0.70)	1.31 (0.62)	1.81 (0.75)	1.96 (0.75)	1.40 (0.62)	2.01 (0.98)	1.64 (0.77)
	>65	1.14 (0.38)	1.67 (0.94)	1.07 (0.25)	2.00 (1.41)	1.33 (0.58)	1.53 (0.51)	1.08 (0.17)	1.24 (0.42)	1.39 (0.61)	1.15 (0.31)
		**Women**					**Men**				
**BAST subscale**	**Education** **< or >HS**	**Mild TBI**	**Moderate-severe TBI**	**Healthy controls**	**Mental Health conditions**	**Other neuro conditions**	**Mild TBI**	**Moderate-severe TBI**	**Healthy controls**	**Mental health conditions**	**Other neuro conditions**
Negative Affect	< HS	3.62 (0.59)	3.59 (0.86)	2.69 (0.65)	3.43 (0.62)	3.37 (0.73)	2.98 (0.70)	2.79 (0.22)	2.60 (0.60)	3.28 (0.66)	3.24 (0.68)
	>HS	3.38 (0.76)	3.30 (0.59)	2.56 (0.63)	3.36 (0.65)	3.24 (0.71)	3.08 (0.72)	3.09 (0.69)	2.50 (0.59)	3.24 (0.72)	3.14 (0.61)
Fatigue	< HS	3.53 (0.87)	3.42 (0.67)	2.50 (0.75)	3.29 (0.80)	3.37 (0.85)	2.70 (0.70)	2.83 (0.29)	2.44 (0.70)	2.90 (0.87)	2.92 (0.72)
	>HS	3.33 (0.90)	3.17 (0.81)	2.43 (0.73)	3.24 (0.80)	3.28 (0.79)	2.96 (0.81)	2.99 (0.70)	2.34 (0.68)	3.05 (0.74)	2.95 (0.63)
Executive Function	< HS	2.28 (0.64)	2.62 (0.62)	2.08 (0.54)	2.41 (0.54)	2.47 (0.60)	2.52 (0.64)	2.30 (0.76)	2.24 (0.56)	2.36 (0.61)	2.44 (0.49)
	>HS	2.21 (0.68)	2.38 (0.76)	1.92 (0.47)	2.22 (0.58)	2.31 (0.58)	2.29 (0.63)	2.41 (0.66)	2.02 (0.49)	2.35 (0.57)	2.32 (0.59)
Impulsivity	< HS	2.33 (0.80)	2.29 (0.33)	1.92 (0.67)	2.25 (0.71)	2.39 (0.72)	2.29 (0.81)	2.33 (0.95)	2.28 (0.77)	2.76 (0.80)	2.68 (0.80)
	>HS	2.21 (0.75)	2.46 (0.66)	1.79 (0.60)	2.21 (0.75)	2.17 (0.73)	2.39 (0.75)	2.55 (0.96)	2.09 (0.67)	2.50 (0.85)	2.52 (0.73)
Substance Abuse	< HS	1.52 (0.69)	1.78 (0.69)	1.32 (0.58)	1.62 (0.81)	1.64 (0.92)	2.20 (1.20)	2.89 (1.35)	1.58 (0.73)	2.14 (1.12)	1.80 (1.02)
	>HS	1.62 (0.85)	2.05 (1.11)	1.25 (0.52)	1.52 (0.77)	1.60 (0.84)	2.05 (0.93)	2.12 (1.07)	1.49 (0.70)	2.05 (1.02)	1.84 (0.95)

*All values are mean (standard deviation) of the average score across items in each subscale*.

*Color values in the tables are to allow for comparison across tables of BAST subscales. Purple, negative affect; Green, fatigue; Blue, executive function; Red, impulsivity; Yellow, substance abuse*.

**Table 3:** Demographics and neurobehavioral symptoms in Spanish-speaking community-dwelling adults with and without TBI.

**Participant characteristics**		**Spanish-speaking national cohort (*****n*** **=** **350)**			
		**Mild-severe TBI** ***n* = 11**	**Healthy controls** ***n* = 296**	**Mental health conditions** ***n* = 27**	**Other neuro conditions** ***n* = 16**
		**n (%)**	**n (%)**	**n (%)**	**n (%)**
Gender	Women	7 (63.6%)	112 (37.8%)	15 (55.6%)	5 (31.3%)
	Men	4 (36.4%)	182 (61.5%)	12 (44.4%)	11(68.8%)
	Transgender/Other	0 (0.0%)	2 (0.6%)	0 (0.0%)	0 (0.0%)
Race	White	7 (63.6%)	179 (60.4%)	15 (55.6%)	5 (31.3)%
	Black/African American	0 (0.0%)	10 (3.3%)	2 (7.4%)	3 (18.8%)
	Asian	0 (0.0%)	2 (0.7%)	0 (0.0%)	0 (0.0%)
	American Indian/ Alaskan Native	0 (0.0%)	2 (0.7%)	0 (0.0%)	2 (12.5%)
	Native Hawaiian/ Pacific Islander	0 (0.0%)	3 (1.0%)	0 (0.0%)	1 (6.3%)
	Other	3 (27.3%)	90 (30.4%)	10 (37.0%)	4 (25.0%)
	Unknown	1 (9.1%)	10 (3.4%)	0 (0.0%)	1 (6.3%)
Ethnicity	Hispanic	9 (81.8%)	280 (94.6%)	27 (100%)	14 (87.5%)
	Non-Hispanic	1 (9.1%)	10 (3.4%)	0 (0.0%)	1 (6.3%)
	Unknown	1 (9.1%)	6 (2.0%)	0 (0.0%)	1 (6.3%)
Education	≤High school	6 (54.5%)	144 (48.6%)	14 (51.9%)	8 (50.0%)
	>High school	5 (45.5%)	152 (51.4%)	13 (48.1%)	8 (50.0%)
		**Mean (SD)**	**Mean (SD)**	**Mean (SD)**	**Mean (SD)**
Age (years)		37.8 (13.4)	38.8 (12.9)	39.1 (10.9)	35.3 (21.5)
Range		18–65	18–80	19–65	18–80
BAST subscales	Negative affect	3.40 (0.64)	2.51 (0.61)	3.21 (0.70)	3.19 (0.55)
	Fatigue	3.38 (1.01)	2.26 (0.72)	2.99 (0.86)	3.02 (0.86)
	Executive function	2.84 (0.84)	2.14 (0.56)	2.31 (0.63)	2.35 (0.50)
	Impulsivity	3.16 (0.68)	1.91 (0.71)	2.23 (0.70)	2.64 (0.93)
	Substance abuse	2.15 (1.08)	1.29 (0.60)	1.38 (0.57)	1.63 (1.15)

*All BAST Subscale values are mean (standard deviation) of the average score across items in each subscale*.

*Color values in the tables are to allow for comparison across tables of BAST subscales. Purple, negative affect; Green, fatigue; Blue, executive function; Red, impulsivity; Yellow, substance abuse*.

**Table 4:** Neurobehavioral symptoms by gender and education among spanish-speakers.

**BAST subscale**	**Women** ***n* = 139**	**Men** ***n* = 209**	**<HS** ***n* = 172**	**>HS** ***n* = 178**
Negative affect	2.71 (0.67)	2.57 (0.33)	2.61 (0.67)	2.63 (0.67)
Fatigue	2.47 (0.84)	2.32 (0.78)	2.41 (0.80)	2.36 (0.82)
Executive function	2.14 (0.59)	2.21 (0.58)	2.31 (0.60)	2.06 (0.54)
Impulsivity	1.92 (0.70)	2.07 (0.80)	1.99 (0.80)	2.03 (0.74)
Substance abuse	1.20 (0.55)	1.43 (0.73)	1.31 (0.67)	1.36 (0.67)

*All values are mean (standard deviation) of the average score across items in each subscale*.

*Transgender/Other not included in table due to low numbers when broken down by injury severity, age, or education*.

*Color values in the tables are to allow for comparison across tables of BAST subscales. Purple, negative affect; Green, fatigue; Blue, executive function; Red, impulsivity; Yellow, substance abuse*.

Cronbach's alphas within each cohort and by health condition, presented in Table 5, indicate that the BAST subscales overall demonstrated acceptable to excellent internal consistency reliabilities across all cohorts and health conditions (α = 0.70–0.92), with a few exceptions falling just below acceptable (α = 0.61–0.69). One notable anomaly for primarily Spanish speakers was the Impulsivity subscale, which had a Cronbach's α = 0.48, potentially due to the very small sample size (*n* = 11) and limited variability in this group. We will evaluate item properties in the future in a larger sample.

**Table 5:** Internal consistency reliabilities of the BAST subscales.

**BAST subscale reliabilities**	**English-speaking (*****n*** **=** **2,511)**				
	**Mild TBI** ***n* = 211**	**Moderate-severe TBI** ***n* = 52**	**Healthy controls** ***n* = 1,548**	**Mental health conditions** ***n* = 427**	**Other neuro conditions** ***n* = 273**
	**α**	**α**	**α**	**α**	**α**
Negative affect	0.90	0.88	0.86	0.88	0.87
Fatigue	0.88	0.75	0.79	0.83	0.78
Executive function	0.88	0.86	0.80	0.81	0.80
Impulsivity	0.73	0.70	0.72	0.73	0.67
Substance abuse	0.77	0.81	0.70	0.76	0.80
**BAST subscale reliabilities**	**Spanish-speaking (*****n*** **=** **350)**				
	**Mild-severe TBI** ***n* = 11**		**Healthy controls** ***n* = 296**	**Mental health conditions** ***n* = 27**	**Other neuro conditions** ***n* = 16**
	**α**		**α**	**α**	**α**
Negative affect	0.81		0.84	0.88	0.69
Fatigue	0.87		0.80	0.81	0.80
Executive function	0.90		0.78	0.83	0.69
Impulsivity	0.48		0.73	0.61	0.75
Substance abuse	0.91		0.73	0.71	0.93

*Spanish-speaking group: TBI, MH, and Other Neuro all below minimum of n = 30 recommended to calculate reliabilities*.

*Color values in the tables are to allow for comparison across tables of BAST subscales. Purple, negative affect; Green, fatigue; Blue, executive function; Red, impulsivity; Yellow, substance abuse*.

### Group Comparisons

#### Gender Differences by Health Condition Within Each Cohort

Table 6 summarizes differences in neurobehavioral symptoms between men and women by health condition within each cohort. Note that most gender differences occurred among English-speakers. Overall, men had significantly higher Substance Abuse and Impulsivity scores while women had higher Fatigue scores.

**Table 6:** Neurobehavioral symptom differences between men and women by health condition in three cohorts.

**Health conditions**		**BAST neurobehavioral subscales**				
		**Negative affect**	**Fatigue**	**Executive function**	**Impulsivity**	**Substance abuse**
English national cohort	Healthy controls			*p* < 0.001^a^	*p* < 0.001^a^	*p* < 0.001^a^
	Mental health conditions		*p* = 0.001^a^		*p* < 0.001^a^	*p* < 0.001^a^
	Other neuro conditions		*p* < 0.001^b^		*p* = 0.001^a^	
	Mild-Severe TBI	*p* < 0.001^b^	*p* < 0.001^b^			*p* = 0.001^a^
Spanish national cohort	Healthy controls					*p* < 0.001^b^
	Mental health conditions					
	Other neuro conditions					
	Mild-severe TBI					

*BAST, Behavioral assessment screening tool. Transgender/Other not included due to small cell sizes. M = Men; W = Women*.

a M > W;

b *W > M*;

*Alpha level for statistical significant set at α = 0.01*.

*Color values in the tables are to allow for comparison across tables of BAST subscales. Purple, negative affect; Green, fatigue; Blue, executive function; Red, impulsivity; Yellow, substance abuse*.

#### Educational Attainment Differences by Health Condition Within Each Cohort

Regarding differences in neurobehavioral symptoms between participants with less than or equal to a High School education and participants with some post-secondary training (>High School) by health condition, healthy controls with some post-secondary training had significantly better Executive Function (*p* < 0.001) in both English and Spanish-speakers. English-speaking healthy controls with some post-secondary training also had fewer Impulsivity symptoms (*p* < 0.001). There were no statistically significant differences in neurobehavioral symptoms by educational attainment in any of the Mental Health, Other Neuro, or TBI groups.

#### Language and Ethnicity Differences by Health Condition

Within the healthy control group, English-speakers had significantly higher scores for Substance Abuse (*p* < 0.001) than did Spanish-speakers; however, within the mild-severe TBI health condition, Spanish-speakers reported significantly higher neurobehavioral symptoms for Executive Function and Impulsivity (*p* < 0.001) than did English-speakers. In the full cohort, combining English- and Spanish-speakers, we ran exploratory analyses to identify any differences within condition based on ethnicity (Hispanic vs. Non-Hispanic) and based on native language and survey language of the participant. The three language groups were English speakers taking the survey in English (E), native Spanish speakers taking the survey in English (SE; confirmed English fluency via responses to open-ended questions), and Spanish speakers reporting in Spanish (SS). Table 7 summarizes the statistically significant findings from all exploratory analyses. We found significant differences in Impulsivity and Executive Function between ethnicity groups in both healthy controls and those with mild-severe TBI. Other notable differences between ethnicity groups included Negative Affect in healthy controls. For all significant comparisons, Hispanic participants reported more frequent symptoms than non-Hispanic participants did. We also observed significant differences between language groups. In healthy controls, native English speakers reported more Fatigue, Impulsivity, and Substance Abuse than primarily Spanish speakers. In those with TBI, primarily Spanish speakers reported more Impulsivity than English speakers did. Native Spanish speakers taking the survey in English did not differ from either other language group in any condition.

**Table 7:** Exploratory group comparisons of ethnicity and language by health condition in a national cohort of adults.

**Health conditions**		**BAST neurobehavioral subscales**					**Ethnicity and language group differences**
		**Negative affect**	**Fatigue**	**Executive function**	**Impulsivity**	**Substance abuse**	
Ethnicity	Healthy controls	*p* < 0.001		*p* < 0.001	*p* < 0.001		Hispanic > Non-hispanic
	Mental health conditions						
	Other neuro conditions						
	Mild-severe TBI			*p* = 0.002	*p* < 0.001		Hispanic > Non-hispanic
Language	Healthy controls		*p* = 0.006		*p* = 0.003	*p* < 0.001	E > SS
	Mental health conditions						
	Other neuro conditions						
	Mild-severe TBI				*p* = 0.003		SS > E

*BAST, Behavioral Assessment Screening Tool. E = Native English Speakers; SE = Native Spanish Speakers reporting in English; SS, Native Spanish Speakers reporting in Spanish. α = 0.01 for Kruskal-Wallace (p-values reported in cells) and α = 0.003 for post-hoc Mann–Whitney U (Language comparisons)*.

*Color values in the tables are to allow for comparison across tables of BAST subscales. Purple, negative affect; Green, fatigue; Blue, executive function; Red, impulsivity; Yellow, substance abuse*.

## Discussion

Practitioners and researchers alike widely accept that neurobehavioral symptoms exist and persist after TBI, but debate still ensues as to symptom etiology. Brain injury itself leads to neurobehavioral problems, but comorbid neurological and mental health conditions, as well as gender, age, and other demographic factors all likely contribute to symptom development and chronicity after TBI as well. Additionally, neurobehavioral symptoms themselves are heterogeneous, incorporating multiple interacting domains, including emotion, cognition, and environmental supports and stressors. To address the variety of factors contributing to multifaceted neurobehavioral symptoms common after TBI and other neurological and mental health conditions, we employed a multidimensional behavioral symptom measure—the BAST—and reported differences in symptom profiles between individuals with various neurological and mental health conditions broken down by gender, age, education, and ethnicity.

The BAST yielded higher scores in TBI than in healthy controls, as expected, which supports its content validity, even in diverse samples. However, individuals with other neurological conditions or with mental health conditions also reported more frequent neurobehavioral symptoms than healthy controls and relatively comparable scores to those with TBI. Therefore, neurobehavioral symptoms common after TBI may be partially due to co-occurring neurological or mental health conditions and are not necessarily attributable to the injury itself.

Similar to past literature in both the general population (29, 31, 32), and after TBI (20, 21), we noted that women reported more fatigue symptoms and men reported more impulsivity and substance abuse symptoms across several health conditions. This suggests that gender affects neurobehavioral symptom reporting independent of the effects of TBI or other health conditions. The symptoms more commonly reported by women are also more common following a mild TBI (24, 48, 56). Given the higher proportion of women with mild vs. moderate-to severe TBI (both in our study and in the broader TBI population), it is difficult to tease out what is specific to injury severity vs. gender.

We did not note the same gender differences in our primarily Spanish speakers, other than men in the healthy control group reporting more substance abuse. This may be due to the small sample sizes, particularly in the TBI, Mental Health, and Other Neuro Conditions, as the descriptive data suggest a gender-based trend similar to those seen in those taking the survey in English. However, the sample size for Spanish-speaking healthy controls was robust, suggesting there may be a cultural component to symptom experience and/or reporting. Gender-associated differences in symptom reporting may be different for primarily Spanish speaking individuals (a proxy measure of level of acculturation) (57) living in the United States (58). The cultural value placed on traditional gender roles, including the expectation that women are submissive and self-sacrificing and that men should be strong, virile, and indomitable in character (59), may contribute to under-reporting of fatigue symptoms in Spanish-speaking women and impulsivity and executive functioning symptoms in Spanish-speaking men living in the United States. The potential association between acculturation and symptom reporting is further supported in the comparisons based on survey language, wherein primarily Spanish-speakers reported less frequent fatigue, impulsivity, and substance abuse symptoms overall than native English-speakers.

The pattern of neurobehavioral symptom reporting by ethnicity group indicated that Hispanic individuals (including both primarily Spanish-speakers and English-speakers) experience more Negative Affect, Executive Function problems, and Impulsivity than non-Hispanic individuals. However, primarily Spanish-speakers reported *fewer* symptoms than native-English speakers did. This may reflect differences in level of acculturation; that is, primarily Spanish-speaking adults living in the United States are likely less acculturated than English-speaking Hispanic adults living in the United States (57). More acculturated individuals more often challenge traditional gender roles, reflecting the majority culture (60), whereas less acculturated individuals are more likely strongly influenced by traditional cultural values. In the absence of culture-based differences in symptom-reporting for more acculturated Hispanic individuals, the negative impact of documented health disparities experienced by Hispanic individuals in the United States (43) may be more evident, as evidenced by the more frequent neurobehavioral symptoms they reported in the present study.

We anticipated that educational attainment would be associated with neurobehavioral symptoms, and we observed differences in Executive Function and Impulsivity, favoring those with some post-secondary education, in healthy controls. The direction of the association between these cognitive characteristics and educational attainment remains unclear. In the general population, lower Executive Function and more Impulsivity convey greater risk for lower educational attainment (61), but post-secondary education may also improve these cognitive functions (62). Despite past research suggesting educational attainment may be protective in the context of TBI, (63) we did not find any differences in neurobehavioral symptoms by education in the TBI or the other health condition groups. It may be that the cognitive consequences of TBI, other neurological conditions, and mental health conditions negate any protective effects of post-secondary education for higher-level cognitive functions. Further, research suggests that the protective effects of education for depression may be most evident in individuals from disadvantaged backgrounds (35, 36) which we did not account for in the educational group comparisons. Further study is warranted to explore how education may be a moderating factor contributing to neurobehavioral symptoms after TBI.

## Limitations

Despite a robust sample size overall, the sample size was small for certain conditions within each cohort, especially when breaking the condition groups down further by gender and age or education. This is especially true for Spanish-speakers with TBI, making inference more difficult. Past literature, especially in TBI, has failed to adequately represent non-binary gender; though we did include Transgender/Other as a gender option, the sample size was too small to include in gender group comparisons or to provide a representative picture of these individuals. Finally, though multiple steps were taken to ensure validity of all collected data, data collected via anonymous survey are prone to bias and error.

## Conclusions

Neurobehavioral symptoms are, indeed, more common in chronic TBI than in a neurologically healthy population. However, these symptoms are also partially attributable to personal factors, like age, gender, and ethnicity, and to health history, including other neurological conditions and mental health conditions. The interactions among cognitive, emotional, personality, biological, and environmental factors contribute to the complexity of neurobehavioral symptoms after TBI. The detailed characterization we present, in English- and Spanish-speaking healthy controls, adults with TBI, and adults with other neurological and mental health conditions from across the United States reveals unique patterns to aid in research on, and clinical interpretation of, neurobehavioral dysfunction after TBI.

## Data Availability Statement

The dataset generated for this study will not be made publicly available. The corresponding author can provide the dataset upon request and execution of the necessary data use agreements.

## Ethics Statement

The studies involving human participants were reviewed and approved by University of Texas Southwestern Medical Center Institutional Review Board. Written informed consent for participation was not required for this study in accordance with institutional requirements.

## Author Contributions

All authors have made significant contributions to the conceptualization, interpretation, and writing of this manuscript and the study described herein and have read and approved the final manuscript. SJ principal investigator on the study presented in this manuscript and was the primary author responsible for writing, conceptualization, and final decisions. AN contributed to data preparation, conducted extensive literature review, and drafted significant portions of the manuscript. LT collaborated with SJ in the initial study conceptualization, provided consultation on all statistical analysis as a statistician, and drafted significant portions of the methods and results.

### Conflict of Interest

The authors declare that the research was conducted in the absence of any commercial or financial relationships that could be construed as a potential conflict of interest.
