# Supplementary material for: Neurobehavioral Symptoms in Community-Dwelling Adults With and Without Chronic Traumatic Brain Injury: Differences by Age, Gender, Education, and Health Condition
Source: Front Neurol. 2019 Nov 20;10:1210. doi: 10.3389/fneur.2019.01210 (PMC6879460; doi:10.3389/fneur.2019.01210)
Supplement: Supplementary file 1 [file Table_1.DOCX]

**Supplemental Table A: Numbers within each cell for Table 2**

| **Age (years)** | **Women**  ***n* = 1040** | | | | | **Men**  ***n* = 1446** | | | | |
| --- | --- | --- | --- | --- | --- | --- | --- | --- | --- | --- |
|  | **Mild TBI** | **Moderate-Severe TBI** | **Healthy Controls** | **Mental Health Conditions** | **Other Conditions** | **Mild TBI** | **Moderate-Severe TBI** | **Healthy Controls** | **Mental Health Conditions** | **Other Neuro Conditions** |
| **18-24** | 24 | 2 | 43 | 40 | 19 | 11 | 3 | 204 | 36 | 31 |
| **25-45** | 45 | 7 | 186 | 109 | 72 | 48 | 14 | 409 | 100 | 73 |
| **46-65** | 38 | 9 | 223 | 65 | 39 | 28 | 9 | 233 | 42 | 15 |
| **>65** | 7 | 2 | 92 | 11 | 7 | 5 | 4 | 152 | 18 | 11 |
| **Education**  **< or > HS** | **Mild TBI** | **Moderate-Severe TBI** | **Healthy Controls** | **Mental Health Conditions** | **Other Conditions** | **Mild TBI** | **Moderate-Severe TBI** | **Healthy Controls** | **Mental Health Conditions** | **Other Conditions** |
| **< HS** | 20 | 6 | 172 | 87 | 58 | 17 | 3 | 290 | 67 | 51 |
| **> HS** | 94 | 14 | 372 | 138 | 79 | 75 | 17 | 708 | 129 | 79 |
